# HeatmapGenerator: high performance RNAseq and microarray visualization software suite to examine differential gene expression levels using an R and C++ hybrid computational pipeline

**DOI:** 10.1186/s13029-014-0030-2

**Published:** 2014-12-24

**Authors:** Bohdan B Khomtchouk, Derek J Van Booven, Claes Wahlestedt

**Affiliations:** Center for Therapeutic Innovation and Department of Psychiatry and Behavioral Sciences, University of Miami Miller School of Medicine, 1120 NW 14th ST, Miami, 33136 FL USA; John P. Hussman Institute for Human Genomics, University of Miami Miller School of Medicine, 1501 NW 10th Avenue, Miami, 33136 FL USA

**Keywords:** RNAseq, Microarray, Heatmap, R programming language, C++ programming language, OpenGL API, Next-Generation Sequencing (NGS), Computational genomics

## Abstract

**Background:**

The graphical visualization of gene expression data using heatmaps has become an integral component of modern-day medical research. Heatmaps are used extensively to plot quantitative differences in gene expression levels, such as those measured with RNAseq and microarray experiments, to provide qualitative large-scale views of the transcriptonomic landscape. Creating high-quality heatmaps is a computationally intensive task, often requiring considerable programming experience, particularly for customizing features to a specific dataset at hand.

**Methods:**

Software to create publication-quality heatmaps is developed with the R programming language, C++ programming language, and OpenGL application programming interface (API) to create industry-grade high performance graphics.

**Results:**

We create a graphical user interface (GUI) software package called HeatmapGenerator for Windows OS and Mac OS X as an intuitive, user-friendly alternative to researchers with minimal prior coding experience to allow them to create publication-quality heatmaps using R graphics without sacrificing their desired level of customization. The simplicity of HeatmapGenerator is that it only requires the user to upload a preformatted input file and download the publicly available R software language, among a few other operating system-specific requirements. Advanced features such as color, text labels, scaling, legend construction, and even database storage can be easily customized with no prior programming knowledge.

**Conclusion:**

We provide an intuitive and user-friendly software package, HeatmapGenerator, to create high-quality, customizable heatmaps generated using the high-resolution color graphics capabilities of R. The software is available for Microsoft Windows and Apple Mac OS X. HeatmapGenerator is released under the GNU General Public License and publicly available at: http://sourceforge.net/projects/heatmapgenerator/. The Mac OS X direct download is available at: http://sourceforge.net/projects/heatmapgenerator/files/HeatmapGenerator_MAC_OSX.tar.gz/download. The Windows OS direct download is available at: http://sourceforge.net/projects/heatmapgenerator/files/HeatmapGenerator_WINDOWS.zip/download.

## Background

Heatmaps are two-dimensional graphical false-color image representations of data using a user-defined color scheme to display values in a data matrix. In the medical research community, heatmaps are used to comparatively illustrate gene expression levels across a number of different samples (e.g., cells under different experimental conditions or samples from different patients) obtained from DNA microarray studies or high-throughput sequencing methods such as RNAseq. Genes that are upregulated (high relative expression value) are colored differently than genes that are downregulated (low relative expression value), thus providing a simultaneous visual representation of gene expression levels across multiple different samples. Heatmaps can be used to, for example, understand what groups of genes are turned on or turned off in various cancer samples as compared to control samples. For instance, heatmaps have been used for gene expression profiling of breast cancer samples to study the differential patterns of gene expression across multiple individual tumors [1].

There are a variety of publicly available and commercial graphical user interface (GUI) software for making heatmaps such as TM4 [2], GenePattern [3], Qlucore [4], and GENE-E [5]. Although there is an abundance of such services, none of them boast comparable graphical capabilities such as those produced by the R programming language: popularly considered to be one of the most powerful and widely used statistical software among researchers in the medical community and the lingua franca for preparing heatmaps for publication. In contrast to previously released heatmap software, HeatmapGenerator is the first GUI heatmap software package capable of compiling heatmaps with just a few button clicks directly from the R language, without making users write a single line of code. In other words, researchers can now use R to make heatmaps without needing any prior programming knowledge. Moreover, the GUI of HeatmapGenerator is the first software of its kind to employ a database storage system that stores a user’s previously generated heatmaps, in case the user wishes to go back to retrieve them from memory or compare them to other generated heatmaps, all within a central repository.

Much of the publicly available heatmap software is not designed for handling gene expression data, instead focusing on an entirely unrelated niche such as geographical interactive online maps [6] or even keyboard layout [7]. Therefore, it is difficult for a medical researcher to find a suitable venue to produce biological heatmaps, instead having to rely on either outsourcing the data to a corporate venue or teaming up with a computational researcher well-versed in computer programming. In this paper, we provide first-time publication of a user-friendly, R-centric GUI for the interactive real-time production of biological heatmaps in the R programming language via software development using the C++ programming language and the OpenGL application programming interface (API).

## Methods

A GUI has been developed to be the frontend to the HeatmapGenerator source code: an R program file designed to create a heatmap. The GUI is written in *FLTK* [8], a cross-platform object-oriented widget toolkit written in the C++ programming language with an interface to OpenGL. OpenGL is the industry standard for rendering cross-platform real-time 2D and 3D vector graphics with frequent interactive applications in flight simulation, scientific visualization, and computer-aided design [9]. C++ is an efficient, compact, fast, portable programming language commonly used in safety-critical applications (e.g., aircraft control systems), operating system development (e.g., Apple Mac OS X, Microsoft Windows), and web search engines (e.g., Google) that links object-oriented programming, procedural programming, and generic programming in one holistic framework [10]. As such, HeatmapGenerator uses C++ to achieve maximal speed, efficiency, and stability when generating heatmap graphics. The source code that creates the heatmaps using the R programming language [11] is executed by calling the Rscript batch interpreter that comes with a standard R download. HeatmapGenerator is able to execute the R heatmap source code within a GUI written in C++/OpenGL using the *FLTK* framework so that the user can press the HeatmapGenerator user-interface buttons (e.g., to change the heatmap’s color scheme) while immediately visualizing these respective changes in the console window without needing to write a single line of code. R must be installed within the local user’s computer, but the GUI uses the R commands of the source code that plot the heatmap, all from a user-friendly clickable interface. The source code utilizes the *heatmap* and *heatmap.2* command [12] within the *gplots* package [13] of the R programming language to ultimately create the heatmap.

### Preliminaries

**Figure 1:**
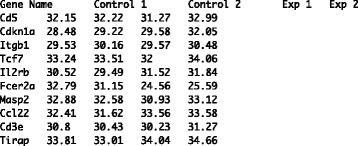
**An example of a textfile for input as a matrix.** A simple exemplary input file to be processed by HeatmapGenerator.

For best use, we refer the reader to the operating system-specific “HeatmapGenerator manual.pdf” file that comes bundled with the HeatmapGenerator download. As stated in this file, to properly use the HeatmapGenerator software package, the user must first download R for (Mac)OSX/Windows at http://cran.rstudio.com/ and preformat the input file to be a tab-delimited textfile (.txt) (an example is provided as EXAMPLE.txt in the HeatmapGenerator download). Specifically, the input file should be a.txt file where each entry is separated by tabs, the first column should contain the item names (e.g., list of gene names), and the other columns should contain numbers corresponding to the respective items. An example of a.txt file table to be imported into HeatmapGenerator is shown in (refer to Figure 1). Note that each column is labeled with its respective header (e.g., Gene Name, Control 1, Exp 1, etc.).

## Results and discussion

**Figure 2:**
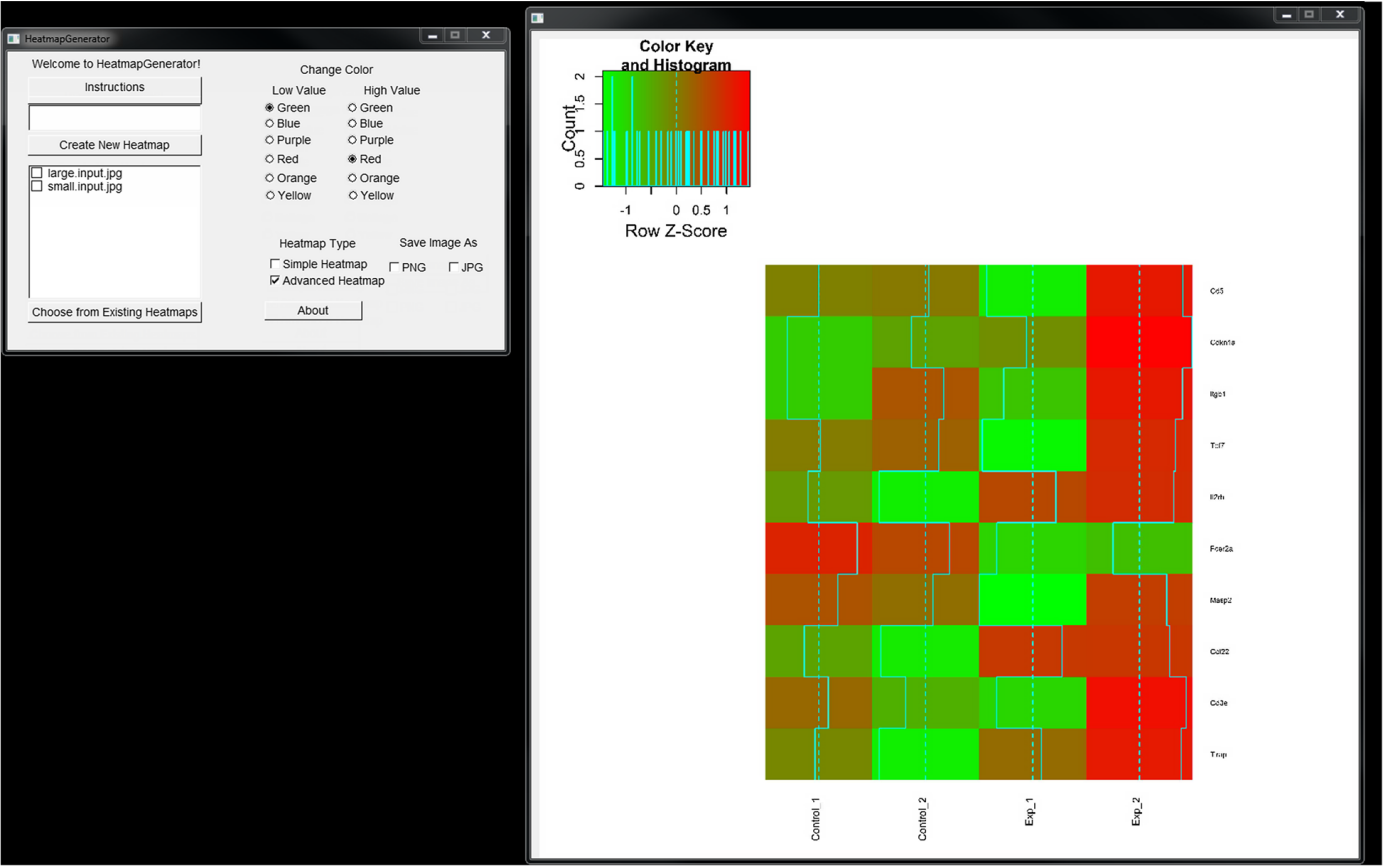
**Small input advanced heatmap generated by HeatmapGenerator.** Advanced heatmap of a small input dataset is created by the graphical user interface of HeatmapGenerator.

**Figure 3:**
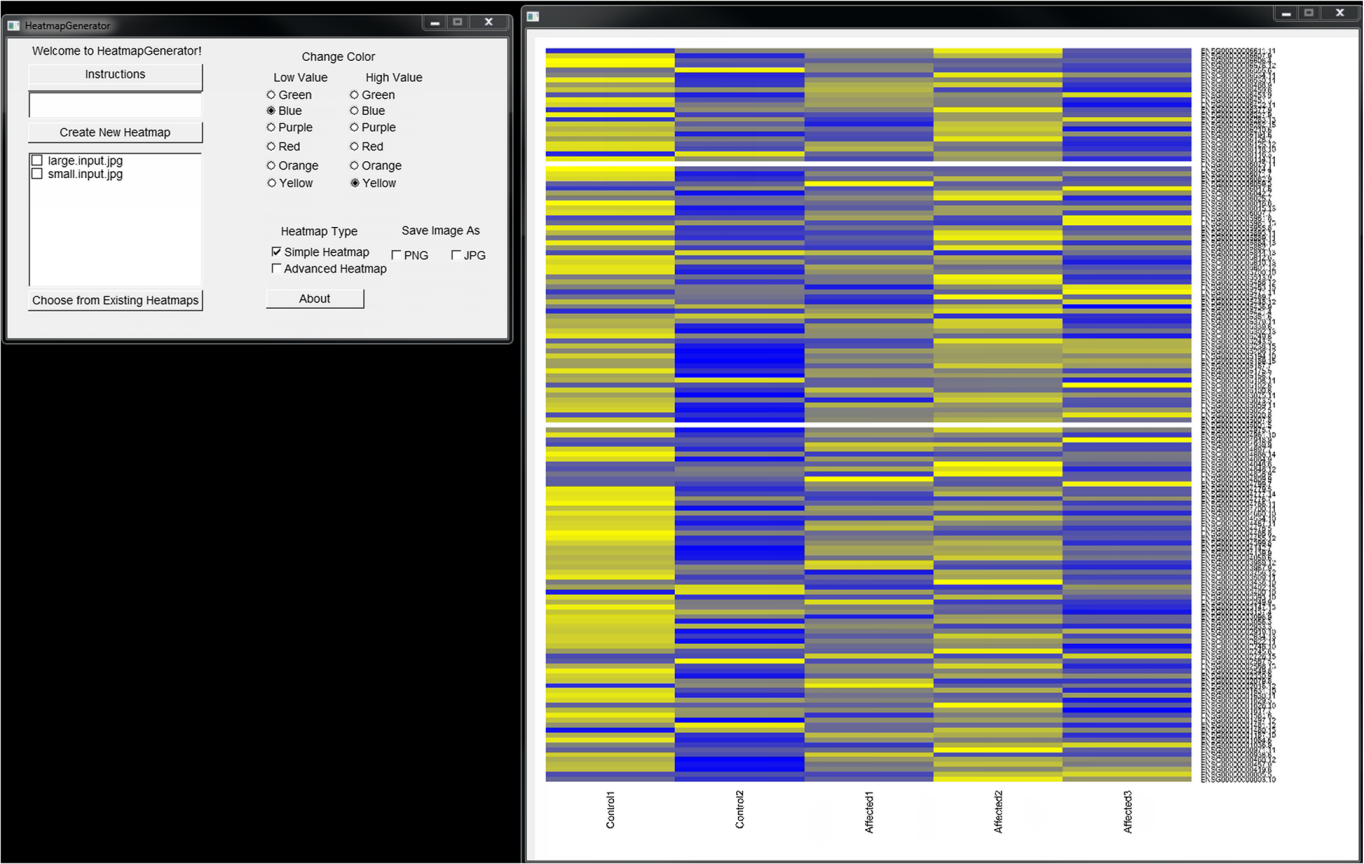
**Large input simple heatmap generated by HeatmapGenerator.** Simple heatmap of a large input dataset is created by the graphical user interface of HeatmapGenerator.

The HeatmapGenerator GUI design gives the user a full suite of options for creating, customizing, saving, storing, and choosing between simple and advanced heatmap construction. Running the HeatmapGenerator software package produces a variety of heatmaps (refer to Figure 2 and Figure 3) showing the relative expression levels of genes from either large or small datasets used as the input to the program. Simple input to the HeatmapGenerator can be as many as a few lines of a textfile up to thousands of lines of data in a textfile that are imported from a large array of analyses.

**Figure 4:**
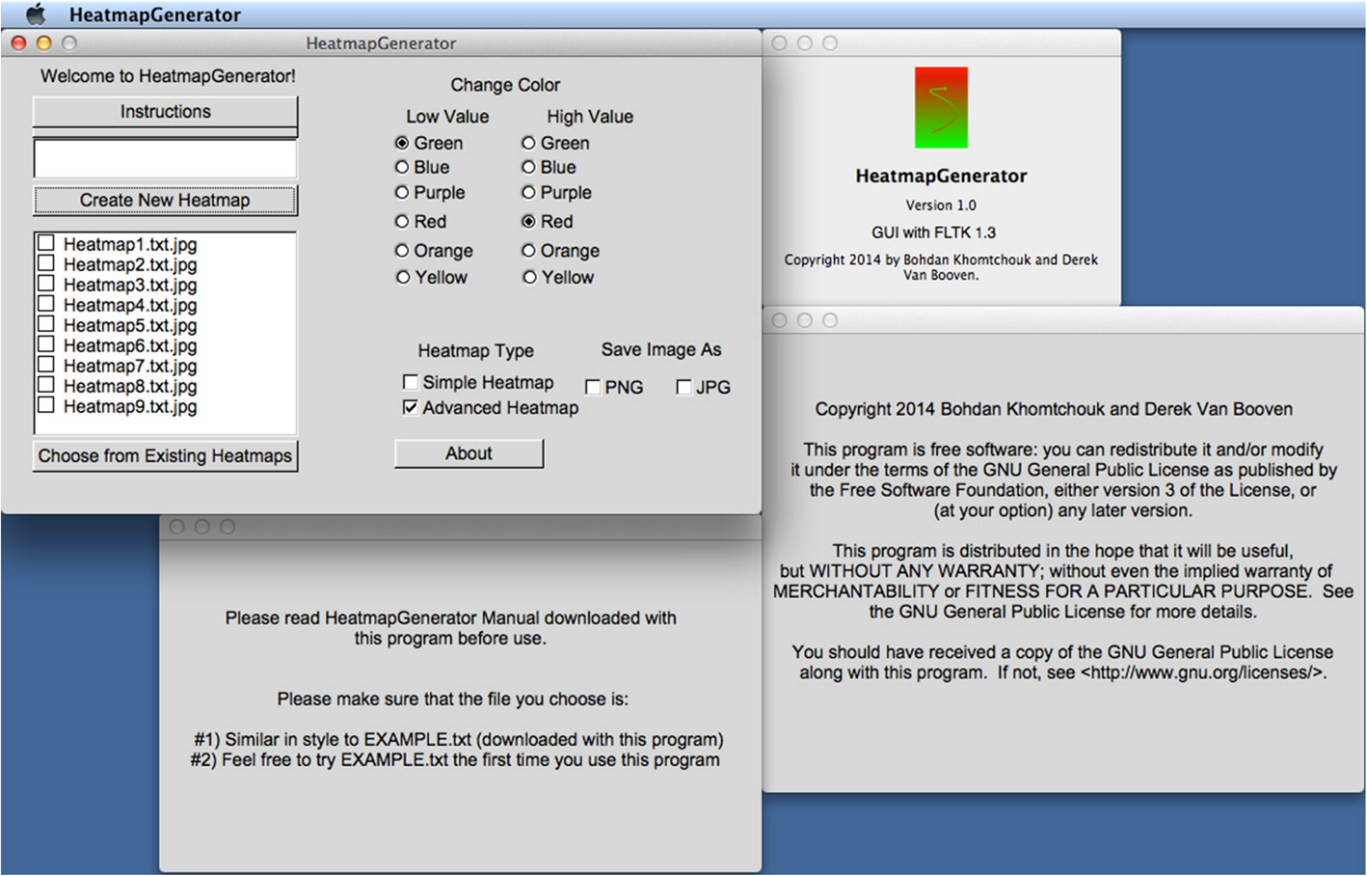
**Database file system of HeatmapGenerator and software information.** HeatmapGenerator can handle an unlimited number of heatmaps in its storage system. Software logo, licensure, copyright information, and user instructions are provided.

Some features of the GUI include a legend that is based on the data. The scaling parameters are developed based on the data that was supplied by the user. Also, the colors of the heatmap can be changed from simple buttons in order to specify a color scheme. After the user has run the HeatmapGenerator, the GUI is able to export the visualized heatmap in either.png or.jpg file formats that allow for publication-quality graphics. A.tiff image is always provided by the software by default. One additional feature is the ability to recall the previous heatmaps that were generated using a backend random access database. The user data is stored in a local database for easy recall and easy reusage to build the same heatmap in case the user wishes to reproduce the file. A user can store an unlimited number of heatmaps in the database file system, provided that these heatmaps reside within the same folder as the HeatmapGenerator program itself (refer to Figure 4).

Changing the color scheme through the simple click of a button changes the colors of the most downregulated and most upregulated genes portrayed in the heatmap. To change the font size of the labels (e.g., the gene names, experiment types) displayed on the heatmap produced, HeatmapGenerator automatically adjusts the size of text labels through its built-in scaling mechanisms. Hence, the user may easily specify modifications to the color selection, text labels, legends, scaling, as well as a local database for storage of previously used heatmaps.

### Applications

One use case for the HeatmapGenerator pertains to RNA next-generation sequencing data (RNAseq). The main goal of RNAseq is to quantify gene expression across multiple biological samples. However, there is no gold standard analysis pipeline for RNAseq data. Rather, there are multiple forms of pipelines that look at the same data, but in two different ways. First, there is a very simplistic model of generating raw counts. These raw counts are generated after global alignments by a program such as HTseq [14]. Second, there is an alternative mathematical approach to calculate a normalized value in order to scale the expression of each gene relative to its respective size (in base pairs). This method is performed by Cufflinks [15], which outputs the normalized value called fragments per kilobase per million (FPKM). The HeatmapGenerator is built to handle both types of data. The user can enter in either raw counts per gene or FPKM per gene, and the HeatmapGenerator will show the differential expression analyses in a visual manner.

## Conclusions

We provide an intuitive and user-friendly GUI software package, HeatmapGenerator, to create high-quality, customizable heatmaps generated using the popular data graphics capabilities of the R programming language. We achieve real-time visualization of these heatmaps using the industry standard high performance graphics of OpenGL. As such, instantaneous responsiveness in heatmap design is achieved through the graphical user interface interaction of a researcher’s input specifications. Furthermore, performance speed and software stability is achieved through the C++ programming language. Moreover, a random access database structure is implemented to reliably store generated heatmaps. The R source code linked to C++ via an OpenGL API allows researchers the ability to create publication-quality biological heatmaps with minimal prior coding experience without sacrificing their desired level of customization: advanced features such as color, legends, text labels, scaling, and database storage can be easily customized with no prior programming knowledge.
